# Acute Necrotizing Pancreatitis—Advances and Challenges in Management for Optimal Clinical Outcomes

**DOI:** 10.3390/medicina61071186

**Published:** 2025-06-30

**Authors:** Ioana Dumitrascu, Narcis Octavian Zarnescu, Eugenia Claudia Zarnescu, Mihai Radu Pahomeanu, Alexandru Constantinescu, Dana Galieta Minca, Radu Virgil Costea

**Affiliations:** 1 Department of General Surgery, “Carol Davila” University of Medicine and Pharmacy, 050474 Bucharest, Romania; ioana.dumitrascu@drd.umfcd.ro (I.D.); eugenia.zarnescu@umfcd.ro (E.C.Z.); radu.costea@umfcd.ro (R.V.C.); 2 Second Department of Surgery, University Emergency Hospital Bucharest, 050098 Bucharest, Romania; 3 Department of Internal Medicine, “Carol Davila” University of Medicine and Pharmacy, 050474 Bucharest, Romania; mihai-radu.pahomeanu@rez.umfcd.ro (M.R.P.); alexandru.constantinescu@umfcd.ro (A.C.); 4 Department of Gastroenterology and Internal Medicine, University Emergency Hospital of Bucharest, 050098 Bucharest, Romania; 5 Department of Public Health, Faculty of Medicine, “Carol Davila” University of Medicine and Pharmacy, 020021 Bucharest, Romania

**Keywords:** acute pancreatitis, pancreatic necrosis, management, drainage, endoscopic necrosectomy, step-up approach

## Abstract

Acute necrotizing pancreatitis (ANP), characterized by necrosis of pancreatic and/or peripancreatic tissues, is a potentially severe and life-threatening complication of acute pancreatitis, exhibiting a considerable mortality rate, particularly in the presence of infection, with rates ascending to 20–30%. Contrast-enhanced computed tomography is the definitive diagnostic standard, although treatment is determined by illness severity and the presence of secondary infection. The management of this condition has undergone considerable evolution, transitioning from initial surgical intervention to a more progressive, minimally invasive strategy. Initial management emphasizes aggressive fluid resuscitation, nutritional support, and monitoring of organ dysfunction. Infected necrosis is a critical factor influencing prognosis and requires intervention, typically starting with percutaneous drainage or endoscopic necrosectomy prior to evaluating surgical debridement. Recent advancements, such as the implementation of endoscopic and minimally invasive techniques, have enhanced outcomes by decreasing morbidity and mortality linked to open surgery. Despite these advancements, optimal treatment strategies are patient-specific and necessitate a multidisciplinary approach. Additional research is necessary to enhance guidelines and optimize patient outcomes.

## 1. Introduction

Acute necrotizing pancreatitis (ANP) accounts for approximately 10–20% of patients and remains one of the most feared complications due to its correlation with high morbidity and mortality [1,2]. Pancreatic necrosis is crucial in both the initiation and exacerbation of the inflammatory response in acute pancreatitis. The breakdown of necrotic pancreatic tissue initiates the production of substantial amounts of proinflammatory mediators, subsequently eliciting an excessive immune response [3,4,5]. The inflammatory response, initially protective, often becomes dysregulated, leading to immunological imbalance and the progression of multiorgan failure [6]. The presence of pancreatic necrosis not only indicates the severity of the disease but also exacerbates it by intensifying the inflammatory response and disrupting immunological balance [7,8].

As the understanding of necrotizing pancreatitis advances, extrapancreatic necrosis (EPN) is emerging as a significant, if previously neglected, aspect of its clinical progression [9]. EPN is characterized by necrosis that extends into the peripancreatic tissues, often associated with an extensive inflammatory response and a severe clinical course. It correlates with increased risks of organ failure, infection, and the need for invasive procedures—all elements that lead to worse outcomes [10]. About one third of cases with necrotizing pancreatitis will develop infection, which is associated with markedly increased morbidity and mortality [11]. Furthermore, several authors documented that the extension of pancreatic necrosis is gradually associated with infection, organ failure, and mortality [12,13]. Nonetheless, although the correlation between pancreatic necrosis and organ failure is acknowledged, the causal relationship has been challenged [14,15]. Consequently, the management of pancreatic necrosis, which includes both prevention and therapy, is of the utmost importance.

In this paper, we aim to review and consolidate the latest standards in conservative and interventional management of acute necrotizing pancreatitis to achieve optimal results.

## 2. Epidemiology

The primary etiological factors of acute pancreatitis are alcohol intake and gallstones, which collectively represent around 70% of cases that can trigger acute necrotizing pancreatitis. Infrequent yet clinically relevant causes encompass hypertriglyceridemia, post-ERCP complications, specific pharmaceuticals, autoimmune disorders, and genetic mutations that influence pancreatic enzyme regulation. The onset of necrotizing pancreatitis, unlike the edematous variant, is believed to stem from an initial and prolonged disruption of pancreatic microcirculation, resulting in ischemia and subsequent acinar cell necrosis [16]. Some individuals are more prone to necrotizing pancreatitis due to a combination of host-specific factors that may intensify the severity of the initial pancreatic injury. This encompasses genetic predispositions, including mutations in PRSS1, SPINK1, or CFTR, which facilitate premature intrapancreatic activation of trypsin [17]. Systemic conditions like obesity can amplify the local inflammatory response via cytokines derived from adipose tissue [18]. Furthermore, pre-existing cardiovascular disease hinders microcirculation, resulting in early pancreatic ischemia and restricting tissue recovery. Consequently, the equilibrium shifts toward necrosis instead of reversible edema.

## 3. Prevention of Pancreatic Necrosis

**Figure 1:**
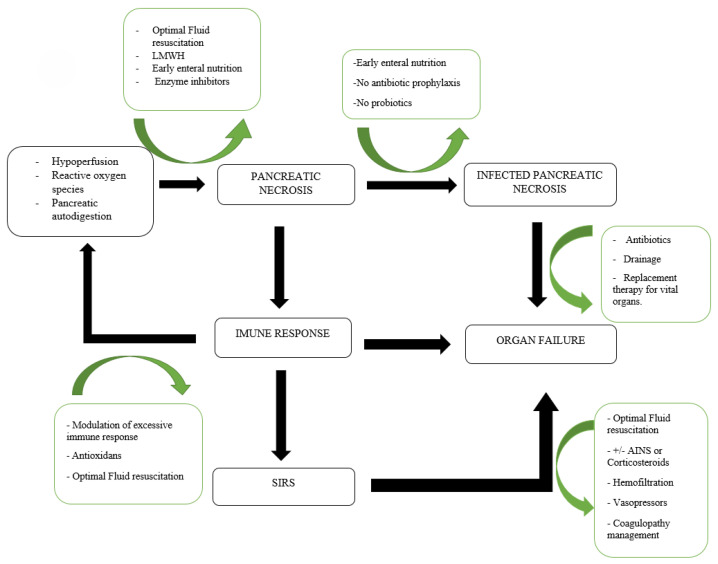
From pancreatic necrosis to organ failure: the role of infection and systemic inflammatory response syndrome (SIRS) and potential therapeutical interventions.

Microcirculatory abnormalities, including vasoconstriction, ischemia, and heightened vascular permeability (see Figure 1), significantly hinder the nutritive perfusion of pancreatic tissues and are crucial in the pathophysiology of pancreatic necrosis during acute pancreatitis [19,20,21]. Moreover, hemorheological alterations, including increased blood viscosity and red blood cell aggregation, lead to stasis and insufficient microvascular perfusion. The end-artery characteristic of pancreatic microcirculation renders it especially susceptible to ischemia damage, leading to tissue hypoxia and necrosis [22]. Hemoconcentration and intravascular coagulation contribute further to the onset of pancreatic ischemia in acute pancreatitis [22,23].

Prompt care of acute pancreatitis may mitigate local consequences such as necrosis. Several critical factors are recognized to avert this result in the early stage of acute pancreatitis, including prompt identification, sufficient intravenous fluid resuscitation, and low-molecular-weight heparin (LMWH), which are important steps that should be undertaken in an effort to prevent pancreatic necrosis [24,25].

### 3.1. Early Recognition and Prediction of Pancreatic Necrosis

In the first four to six weeks after the onset of symptoms, the first type of necrotic collection that surfaces is acute necrotic collection, where the early recognition of those lesions is important. The 2024 American College of Gastroenterology (ACG) guidelines on acute pancreatitis management advises CT scans within the initial 48–72 h only for patients who exhibit no clinical improvement or when the diagnosis is uncertain [26]. Consequently, endoscopic ultrasound (EUS) [27,28,29] or contrast-enhanced ultrasound (CEUS) [30] holds significant utility during this period. EUS can accurately identify pancreatic necrosis and associated conditions such as common bile duct stones; however, it is more intrusive and less accessible at smaller centers [31]. An effective approach may involve monitoring the patient using CEUS, as a prior original work by Golea et al. demonstrated that CEUS exhibited a 62.5% concordance in detecting areas of pancreatic necrosis when compared to CT scans [32]. An original study conducted in China demonstrated a robust connection between the CT Severity Index (CTSI) and the US Severity Index (USSI), with a sensitivity of 90% and specificity of 95% for CEUS in diagnosing pancreatic necrosis [33].

Pancreatic necrosis is an important factor influencing the severity of acute pancreatitis, as patients with both sterile and infected necrosis exhibit similar probabilities of developing organ failure [12,34,35,36]. Consequently, we can use validated and innovative severity prediction systems utilized in acute pancreatitis (see Table 1) [37,38,39,40]. Individually, they may lack high reliability; nevertheless, collectively, they could serve as cost-effective methods to anticipate severe disease and necessitate enhanced imaging surveillance for the early diagnosis of pancreatic necrosis.

Recently, a team lead by Song created a nomogram-based model that demonstrates good results in predicting infected pancreatic necrosis in cases of moderately severe and severe acute pancreatitis [53]. This model is based on the following five variables: platelet count, hematocrit, albumin-to-globulin ratio, severity of acute pancreatitis, and mCTSI score. Despite the model’s validity across three cohorts (training, validation, and external validation), it remains essential to replicate this model in additional populations.

### 3.2. Adequate Intravenous Fluid Resuscitation

Acute pancreatitis involves significant endothelial damage and increased vascular permeability, leading to fluid loss and potential thrombus development, resulting in parenchymal ischemia [54,55,56]. The objective for intravenous hydration is to correct hypovolemia and sustain sufficient tissue perfusion to avert ischemia and cellular death, which could, ultimately, result in pancreatic necrosis.

Crystalloid solutions are recommended [26,57], particularly lactated Ringer’s solution, due to its resemblance to plasma in electrolyte composition, its capacity to restore oncotic pressure and albumin levels, and prevention of organ failure. The anti-inflammatory properties of lactated Ringer’s solution in managing acute pancreatitis, is evidenced by a reduction in C-reactive protein levels and incidence of SIRS [58,59].

The prompt initiation of treatment following the development of acute pancreatitis should correspondingly influence the outcomes due to the potentially rapid progression of the condition. A prospective study demonstrates that timely intervention is crucial, since early commencement (within 2 h) of vigorous fluid therapy may be beneficial; but, if delayed by 6–8 h post-admission, the risk of persistent organ failure and mortality may worsen [60]. Following initial findings [61] indicating no advantage of early Ringer treatment over normal saline, a subsequent multi-institutional investigation [62] with 999 patients established that the administration of lactated Ringer’s solution within the first 24 h of hospitalization correlated with improved acute pancreatitis severity.

Numerous international guidelines highlight the significance of early, goal-directed fluid resuscitation in acute pancreatitis, though specific recommendations differ. There is a consensus on the importance of targeting clinical and biochemical parameters, including heart rate, mean arterial pressure, urine output, and hematocrit levels, to inform therapeutic decisions. The International Association of Pancreatology [63] and the American College of Gastroenterology [26] support fluid administration rates of 5–10 mL/kg/h or 250–500 mL/h, respectively. In contrast, the Italian [64] and Japanese [65] guidelines propose initial boluses followed by maintenance infusions adjusted according to patient response. Despite differences in fluid rates and volumes, guidelines agree on the importance of early initiation, careful monitoring, and individualized adjustments to mitigate complications associated with both under- and over-resuscitation.

Despite an initial enthusiasm regarding aggressive resuscitation, it was later demonstrated that this strategy can be associated with detrimental results. In the early phase, a randomized controlled trial (WATERFALL trial), conducted by de-Madaira et al., evaluating aggressive fluid administration strategy (20 mL per kilogram of body weight, followed by 3 mL/h/kg) vs. moderate resuscitation with lactated Ringer’s solution was discontinued due to safety concerns, as 20.5% of patients experienced fluid overload, with no statistically significant difference in severity observed [66]. A meta-analysis including six randomized controlled trials with a total of 632 patients established that aggressive fluid resuscitation correlates with increased mortality (RR: 2.40 and CI: 1.38–4.19) in comparison to moderate fluid replacement in individuals with acute pancreatitis, with no significant differences observed in the incidence of organ failure, severe pancreatitis, pancreatic necrosis, clinical improvement, development of systemic inflammatory response syndrome (SIRS), persistent SIRS, or duration of hospital stay [67].

A meta-analysis of eight trials including 557 patients demonstrated a 31% reduction in the incidence of moderate to severe acute pancreatitis and a 62% reduction in mortality risk [68]. Lactated Ringer’s solution was linked to a markedly reduced likelihood of requiring intensive care (RR: 0.50), organ failure (RR: 0.78), and local complications (RR: 0.64). However, no statistically significant link was found between Lactated Ringer’s solution fluid therapy and the development of necrosis (RR: 0.70, 95% CI: 0.40–1.23, *p* = 0.176). A recently published meta-analysis of six randomized controlled trials and four observational studies, encompassing a total of 1500 acute pancreatitis patients, compared lactated Ringer’s solution to saline fluid resuscitation [69]. The authors showed that lactated Ringer’s solution is associated with a markedly diminished risk of moderate to severe acute pancreatitis, a reduced duration of hospitalization, a lower rate of ICU admissions, and a decreased occurrence of local complications. Moreover, subgroup analyses suggest Lactated Ringer’s solution’s capacity to mitigate pancreatic necrosis.

### 3.3. Low-Molecular-Weight-Heparin

As previously stated, changes in microcirculation in the pathophysiology of pancreatic necrosis are recognized to diminish pancreatic blood flow. Consequently, the use of low-molecular-weight heparin (LMWH) was suggested to mitigate necrosis and restore perfusion to the pancreatic tissue [70]. At the same time, due to the heightened risk of both spontaneous [71,72] and procedure-related [73,74] hemorrhage in patients with pancreatic/peripancreatic necrosis, anticoagulation has historically been administered with considerable caution.

Considering all these factors, the safety and efficacy of LMWH treatment for patients with acute pancreatitis were evaluated (see Table 2). A 2009 study conducted on patients with severe acute pancreatitis demonstrated that the addition of low-molecular-weight heparin significantly reduced the CT score of pancreatic necrosis (CTSPN) compared to standard therapy alone [75]. A randomized-controlled trial conducted by Tozlu demonstrated that administering 1 mg/kg b.i.d. for 7 days resulted in a significant reduction in pancreatic necrosis cases, decreased local and systemic complications, and no variation in hemorrhagic complications; however, it did not enhance mortality rates in moderately severe and severe cases [24]. A phase 3 randomized controlled trial in India demonstrated that the addition of Enoxaparin 1 mg/kg twice daily during hospitalization considerably reduces the incidence of pancreatic necrosis, with no adverse events recorded [76]. A recently published prospective study (20) demonstrated that the use of LMWH into conventional treatment for severe acute pancreatitis considerably reduces the CTSPN and death rate [77].

Despite the fact that anticoagulants are not advised in contemporary clinical guidelines [26,63,78,79], several meta-analyses indicate that early management with low-molecular-weight heparin is safe and may enhance the prognosis of non-mild acute pancreatitis regarding organ failure, duration of hospitalization, and mortality rates [80,81,82].

**Table 2:** Summary of studies evaluating the effect of low-molecular-weight heparin in acute necrotizing pancreatitis.

Author, Year	I/C *	N	Male, n (%)	Age, Mean (Years)	APACHE II on Admission	Dose, T/P ^#^	D ^†^	Vascular Thrombosis	GI Bleeding	Need for Surgery	Organ Failure	Shock	Mortality	Length of Hospital Stay
Patil 2022 [76]	I	70	37 (52.9%)	44.2 ± 16.7	NA	T	<8 days	1 (1.4%)	NA	NA	12 (17.1%)	NA	0 (0%)	10.9 ± 1.7
	C	70	40 (68.6%)	40.5 ± 15.7	NA			9 (12.9%)	NA	NA	14 (20.0%)	NA	3 (4.35%)	11.5 ± 2.9
Kumbha 2022 [83]	I	50	42 (84%)	NA	NA	T	<8 days	NA	NA	NA	23 (46%)	NA	1 (2%)	NA
	C	50	41 (82%)	NA	NA			NA	NA	NA	14 (28%)	NA	1 (2%)	NA
Kröner 2021 [84]	I	5776	3089 (53.5%)	65.52 ± 16.91	NA	NA	NA	NA	NA	NA	1361 (23.6%)	239 (4.8)	141 (2.4%)	5.0 ± 39.1
	C	5776	3068 (53.1%)	65.22 ± 16.59	NA			NA	NA	NA	1495 (25.9%)	279 (4.8)	188 (3.3%)	4.0 ± 30.6
Vadlamudi 2021 [85]	I	290	NA	NA	NA	NA	NA	1 (0.3%)	1 (0.3%)	NA	NA	NA	NA	NA
	C	99	NA	NA	NA			0	0	NA	NA	NA	NA	NA
Zhou 2020 [86]	I	169	88 (52.1%)	42.6 ± 5.05	9.0 ± 1.01	T	NA	36 (21.3%)	25 (14.8%)	NA	40 (23.6%)	37 (21.9)	22 (1.2%)	30 ± 6.33
	C	104	65 (62.5%)	42.5 ± 2.33	9 ± 0.6			48 (46.1%)	20 (19.2%)	NA	37 (35.5%)	33 (31.7)	23 (22.1%)	38 ± 6.91
Tozlu 2018 [24]	I	50	22 (44%)	51 ± 16	NA	T	<8 days	1 (2%)	NA	NA	6 (12%)	NA	0 (0%)	7.8 ± 3.4
	C	50	24 (48%)	52 ± 20	NA			7 (14%)	NA	NA	21 (42%)	NA	5 (10%)	11.8 ± 12.5
Du JD 2014 [87]	I	68	35 (51.5%)	50.9 ± 0.25	9.27 ± 0.14	P	8–14 days	NA	NA	2 (2.9%)	4 (5.9%)	NA	1 (1.5%)	18.15 ± 2.35
	C	66	34 (51.5%)	50.5 ± 1.0	9.18 ± 0.03			NA	NA	5 (7.6%)	10 (15.1%)	NA	5 (7.6%)	23.25 ± 4.15
Lu 2009 [75]	I	135	84 (62.2%)	56 ± 11	11.5 ± 3.6	P	<8 days	0	8 (5.9%)	6 (4.4%)	19 (14.1%)	9 (6.6)	14 (10.4%)	30 ± 8
	C	130	72 (55.4%)	54 ± 10.8	5.4 ± 3.4			1 (0.7%)	9 (6.9%)	15 (11.5%)	34 (26.1%)	7 (5.4)	40 (30.6%)	46 ± 11

* I = Intervention/C = Control; ^#^ T = Therapeutic/P = Prophylactic; ^†^ D = Duration.

### 3.4. Epidural Anesthesia

Epidural analgesia is frequently employed during major surgeries, postoperatively in some intensive care unit patients, and has also been utilized to manage pain in patients with acute pancreatitis [88]. In animal studies, epidural analgesia facilitated partial restoration of microcirculatory pancreatic flow, prevented the development of pancreatic necrosis, and led to reduced histopathological tissue damage [89,90]. A single-center randomized experiment demonstrated that thoracic epidural analgesia enhanced pancreatic perfusion on computed tomography (43% vs. 7%, *p* = 0.0025), accompanied by a nonsignificant reduction in intubation requirements compared to a control strategy lacking epidural analgesia [91].

Jabaudon et al. undertook a multicenter, open-label, randomized, and controlled trial (EPIPAN study) with 148 patients with acute pancreatitis [92]. The number of ventilator-free days and the incidence of abdominal and extra-abdominal problems were comparable between the groups; hence, this investigation did not demonstrate the anticipated advantage of epidural analgesia alongside standard treatment. A recent meta-analysis including five randomized controlled trials involving 260 patients with acute pancreatitis indicated that opioid requirements were markedly reduced; however, there was no significant difference in in-hospital mortality, mechanical ventilation, sepsis events, or duration of hospital/ICU stay [93]. Consequently, additional high-quality, large-scale randomized trials are required to elucidate the potential advantages of EA in the management of acute pancreatitis.

## 4. Prophylaxis of Infection of Pancreatic Necrosis

### 4.1. Nutrition

The majority of acute pancreatitis cases recover spontaneously within 48 h and do not require nutritional support. However, 15–20% of patients have a hypercatabolic condition, making nutritional support essential for delivering calories, mitigating complications, and enhancing outcomes. Metabolic expenditure rates are exceedingly elevated, and nutrient depletion may ensue rapidly unless nutritional supplementation is initiated.

Nutritional therapy for acute pancreatitis has traditionally been based on the notion of resting the gut to prevent any stimulation of pancreatic exocrine production. However, it was demonstrated that the secretion of pancreatic juice and trypsin is diminished during acute pancreatitis, resulting in a state of unresponsiveness in the pancreas [94]. In the same time, bacterial gut translocation has been documented in both animal models [95] and clinical studies [96,97], frequently associated with acute pancreatitis, and evidence suggests a correlation with patient outcomes, particularly in cases of necrotizing acute pancreatitis. Therefore, the current paradigm has shifted toward enteral nutrition to emphasize reducing pancreatic stimulation to basal levels while preserving gut integrity and immune modulation to minimize the risk of multiorgan failure, infections, and mortality [98,99,100].

Despite initial concerns, total enteral nutrition for preventing pancreatic necrosis infection in severe acute pancreatitis exhibited superior outcomes compared to total parenteral nutrition (TPN) [101,102,103], thereby reinforcing the assertion that early enteral nutrition is more efficacious than delayed enteral nutrition [104]. Conversely, Bakker et al. conducted a large randomized trial involving 208 patients with predicted severe acute pancreatitis and discovered that early enteral tube feeding within 24 h did not diminish the infection rate in comparison to on-demand feeding [105]. The nasogastric route is chosen over the nasojejunal route for enteral feeding due to similar safety and efficacy [106,107,108]. Parenteral nutrition should be utilized only when the enteral route is infeasible, not tolerated, or insufficient to meet caloric requirements [109].

### 4.2. Antibiotics

Preventing infection is a crucial aspect of managing pancreatic necrosis. Numerous studies (see Table 3) and meta-analyses have demonstrated a lack of evidence to endorse the routine administration of antibiotic prophylaxis in individuals with severe acute pancreatitis [110,111,112]. Furthermore, these studies have illustrated the detrimental consequences of antibiotic prophylaxis, including a heightened prevalence of multi-drug-resistant bacteria and a rise in fungal infections, thereby elevating mortality and morbidity rates [113]. The Surviving Sepsis Campaign (2021) advocates against prolonged systemic antimicrobial prophylaxis in individuals with severe inflammatory conditions of non-infectious origin to bolster the prior suggestion [114]. Unfortunately, numerous studies worldwide have demonstrated inadequate compliance with standards in practical settings [115,116,117].

## 5. Pancreatic Necrosis Treatment

### 5.1. Antibiotics

Timely recognition of infection and prompt administration of antibiotics are essential for improving patient outcomes. Procalcitonin levels have been investigated to predict the advantages of including antibiotics in a patient with acute pancreatitis [125,126]. Bacterial sepsis can be distinguished from systemic inflammation by procalcitonin algorithms. The PROCAP study demonstrated that procalcitonin-guided management can decrease antibiotic utilization without elevating the risk of infection or injury in individuals with acute pancreatitis [127]. Blood metagenomic next-generation sequencing (mNGS) might represent a significant advancement in the early diagnosis of infected pancreatic necrosis. A prospective multicenter clinical investigation in China assessed infected pancreatic necrosis against sterile pancreatic necrosis utilizing the mNGSC technique, procalcitonin levels, and blood cultures in 78 patients with febrile acute necrotizing pancreatitis [128]. In comparison to procalcitonin and blood culture, mNGS exhibited a markedly superior sensitivity rate (86.7% vs. 56.7% vs. 26.7%, *p* < 0.001). Otherwise, infected pancreatic necrosis should be suspected when imaging shows gas within the pancreas or in the peripancreatic collection. Additional indicators of infected necrosis could include fever, bacteremia, exacerbated leukocytosis, or clinical deterioration.

Current guidelines advocate for the utilization of carbapenems, quinolones, and metronidazole, recognized for their ability to penetrate pancreatic necrosis [26,63,129]. These interventions may be beneficial in postponing or occasionally avoiding surgery, perhaps decreasing morbidity and death in individuals with infected pancreatic necrosis. The length of antibiotic treatment is dictated by the severity of the infection, clinical response, and imaging results, with a minimum duration of 14 days; nevertheless, the choice to prolong or terminate antibiotics should be individualized for each patient. There are still no clear recommendations regarding the use of probiotics to modulate the intestinal microbiome during severe acute pancreatitis [130].

### 5.2. Interventional Treatment

#### 5.2.1. Indications and Optimal Timing for Intervention

Patients with sterile acute necrotizing pancreatitis frequently respond favorably to conservative treatment, resulting in a substantial number recovering without surgical or endoscopic interventions. Conversely, infected cases typically necessitate invasive intervention for successful resolution. Several pivotal inquiries emerge concerning the interventional care of acute necrotizing pancreatitis, as follows: indications, timing, and modality to perform the procedures.

Multiple indications (see Table 4) exist for the procedural management of acute necrotizing pancreatitis, which may be executed via endoscopy, percutaneous methods, or surgical intervention. The primary objectives of managing necrotizing pancreatitis are debridement, infection control, evacuation of fluid and solid necrotic material, and addressing complications arising from necrosis.

The optimal timing for intervention in necrotizing pancreatitis is a topic of considerable research and discussion, due to the disease’s evolving characteristics [131,132]. Effective management requires balancing early supportive care with prompt interventions to reduce complications and minimize procedural risks. Current protocols for managing necrotizing pancreatitis advise delaying drainage or necrosectomy for a minimum of four weeks after the initial presentation to permit the development of a well-defined capsule around necrotic collections, thus enabling safer and more effective intervention [26,63,64,79]. According to the updated Atlanta criteria, pancreatic necrotic collections typically necessitate more than four weeks for full encapsulation [133]. This process, along with liquefaction, improves the delineation between necrotic and viable tissue, thereby enabling a more accurate and regulated intervention thereafter. Thus, postponed intervention is frequently more advantageous than early intervention owing to its enhanced feasibility and safety.

However, there are cases where there is compelling evidence of infection accompanied by clinical deterioration despite optimal supportive care, suggesting that conservative treatment may not be applicable to all patients with infected pancreas necrosis [134]. Consequently, an early intervention—before the four-week threshold—may be essential to avert additional problems and enhance patient outcomes. Definitions of early necrosectomy encompassed various cut-off values—15 days [135], 10–12 days [136], or 48–72 h [137]—with most demonstrating dismal outcomes from early intervention. A meta-analysis of 11 retrospective studies involving 775 patients with early therapies and 725 patients with delayed interventions indicated greater mortality rates associated with early interventions for necrotizing pancreatitis [138].

The implementation of minimally invasive procedures (endoscopic [139,140], percutaneous [141,142], or surgical [143,144]) marked a pivotal advancement in the treatment of these individuals aiming for better results comparative to open necrosectomy (see Table 5). In a global poll of experienced pancreatologists, 45% indicated that they advocate for rapid catheter drainage upon the diagnosis of infected pancreatic and peripancreatic necrosis, whereas 55% of respondents prefer to defer intervention and monitor the efficacy of antibiotics [145].

A retrospective study on acute necrotizing pancreatitis compared 76 patients who underwent early intervention (less than four weeks) with 117 patients who received standard intervention [150]. Seventy-five percent of the interventions involved endoscopic drainage with or without necrosectomy. The findings indicate that early intervention does not result in a higher incidence of complications and yields comparable improvements in organ function relative to delayed intervention; however, it may be linked to a slightly increased probability of necessitating subsequent surgical procedures. Comparable outcomes were observed in the POINTER trial, conducted by the Dutch Pancreatitis Study Group, which compared immediate catheter drainage (within 24 h post-randomization upon diagnosis of infected necrosis) to delayed catheter drainage (once walled-off necrosis was established) [156]. This study did not observe substantial disparities in complications and mortality; nevertheless, it appears that patients who underwent delayed catheter drainage necessitated fewer later procedures. The long-term follow-up of the same population indicates that the delayed-drainage strategy utilizing antibiotics in patients with infected necrotizing pancreatitis leads to fewer interventions than rapid drainage, therefore making it the preferred method [162].

Establishing the ideal timing for intervention in necrotizing pancreatitis necessitates a patient-centered strategy that meticulously weighs the disease’s progression against the potential procedural risks.

#### 5.2.2. Minimally Invasive Approaches

Multiple techniques for the treatment of acute necrotizing pancreatitis utilizing minimally invasive approaches have been described [163]. These can be categorized based on the access route as transperitoneal, retroperitoneal, or per-oral transmural and by the type of visualization method as laparoscopic, endoscopic, or radiologic. All the aforementioned can be utilized as distinct or integrated methods.

##### Percutaneous Drainage

An essential method for managing necrotizing pancreatitis is image-guided percutaneous catheter drainage (PCD). Often the primary choice, it has demonstrated efficacy as a standalone treatment in approximately 50% of instances [164]. It aids in enabling minimally invasive necrosectomy and is significant in dual-modality therapy involving endoscopic drainage.

Percutaneous drainage catheters may be inserted by a peritoneal or retroperitoneal approach, with the retroperitoneal route favored for its reduced risk of peritoneal contamination and potential to lessen bowel injury, contingent upon the selected access method. Computed tomography is more frequently used over ultrasound for guidance due to its enhanced vision of adjacent structures, hence minimizing procedure risks [165,166,167]. Moreover, CT facilitates prompt post-procedural evaluation, assisting in the assessment of catheter effectiveness and the possible necessity for further drainage. The size of the catheter is a crucial determinant affecting the efficacy and results of percutaneous catheter drainage; nevertheless, it is insufficiently assessed in the literature. Contemporary experience indicates that small-bore catheters (8–12 F) are appropriate for liquid collections with limited solid debris, but more structured, debris-rich collections may necessitate larger bore catheters [153]. One must consider that the larger bore catheters could be associated with a heightened risk of problems, including injury to the intestines, solid organs, and blood vessels [168].

Various advanced procedures have been proposed to improve the effectiveness of percutaneous drainage in the treatment of pancreatic fluid collections. The kissing catheter approach entails the insertion of two catheters through a single puncture site after serial tract dilation, with one serving as a lavage line for irrigation and the other as a suction catheter to enhance drainage efficiency and treatment results [169]. An alternative sophisticated method is the double-lumen catheter, which integrates both a flushing (inlet) and a drainage (aspirator) catheter into a larger tube equipped with many lateral holes [170]. This arrangement optimizes fluid exchange, improves drainage efficiency, and reduces the likelihood of catheter blockage, providing an advanced approach for percutaneous management of pancreatic collections.

Adjunctive approaches have been investigated to improve the effectiveness of percutaneous catheter drainage in the treatment of pancreatic fluid collections. Extensive lavage with normal saline has shown advantages in ameliorating organ failure and lowering APACHE II scores, although it does not markedly affect the incidence of new-onset organ failure, catheter-related problems, or the necessity for catheters [171]. No studies have dealt with the application of local antibiotic instillation via percutaneous catheters, yet its potential to enhance outcomes in PCD appears promising [172]. Moreover, necrolytic drugs like streptokinase have been shown to facilitate drainage by boosting fibrinolysis, resulting in enhanced sepsis resolution and decreased rates of surgical intervention [173].

Complications of percutaneous catheter drainage include secondary infections, bleeding, and external pancreatic or enteral fistulas [174]. Hemorrhage, usually of venous origin, can lead to hemodynamic instability and may necessitate active interventional treatment [175]. Pancreatic istulae typically resolve with conservative treatment; however, persistent instances may require pancreatic duct stenting [176]. Gastrointestinal fistulae, typically seen near the splenic flexure, may be treated conservatively or surgically based on their severity, especially when accompanied by bleeding or sepsis [177].

##### Endoscopic Interventions

Since the first report of direct endoscopic necrosectomy [178], transmural endoscopic drainage has emerged quickly among percutaneous methods, as the appropriate first-line, nonsurgical techniques for addressing pancreatic wall-off necrosis [129,179,180]. Although different approaches for the effective management of wall-off necrosis (WON) are available, the endoscopic technique appears to result in fewer procedure-related adverse effects and has become an essential technique in this field [181,182]. There is general agreement that endoscopic necrosectomy is a safe and successful procedure with acceptable mortality and morbidity rates when conducted in expert centers [181,183].

Endoscopic intervention on symptomatic or infected WON attempts to access the collection and its drainage in the digestive lumen (stomach or duodenum) by means of metal or plastic stents. The location of transmural puncture may be readily identified by a bulge in 40–60% of patients [184]. If the bulge is not visible, endoscopic ultrasound (EUS) is used to identify the collection, evaluate its contents, and direct the puncture, therefore reducing the risk of injury to adjacent structures [185,186].

The success rate (technical success, the number of endoscopic sessions, the requirement of percutaneous drainage, long-term success, and recurrence) for endoscopic draining of WON was reported to be around ninety percent [187,188,189]. Nonetheless, early interventions [155], collections over 10 cm, those extending into the paracolic gutters [179], and collections with a solid debris percentage of 30% or above [188,189] are less likely to resolve by draining alone and often need further interventional management.

There are two categories of stents available, as follows: plastic and metal. A double pigtail plastic stent may be used as a single transluminal gateway approach or a multiple transluminal gateway technique for collections beyond 12 cm, using EUS guidance, but with a reduced success rate. The use of several double pigtail stents reduces the likelihood of occlusion or migration [190]. Despite being an economical and accessible choice, double pigtail stents have a small diameter that limits effective drainage of large debris.

Consequently, the alternative to these stents is totally covered self-expandable metal stents, which possess a substantial diameter that facilitates necrosectomy via the stent itself. Their drawbacks include stent migration, hemorrhage from the created fistulous tract, and migration leading to perforations or gastrointestinal bleeding [191,192]. Lumen-apposing metal stents (LAMS), characterized by a dog-bone shape, have the advantage of inhibiting stent migration, facilitating the apposition of the walls of two luminal structures, therefore establishing communication and avoiding leakage from the fistula [193]. It also permits access of the endoscope via its lumen to the WON for necrosectomy. Among the risks associated with this type of stent, bleeding and perforation are the most severe, since they may affect both the gastrointestinal wall and the retroperitoneum [194]. A significant consequence is the fibrotic response induced by the stent, which may result in compression of the common bile duct or buried stent syndrome [195]. After placement, early removal of LAMS at 3 weeks post-intervention may be proposed if the WON is resolved on sectional imaging examination [196].

Direct endoscopic necrosectomy (DEN) is indicated mainly for patients with pancreatic necrosis who inadequately respond to large-bore self-expanding metal stents, lumen-apposing metal stents, or plastic stents with irrigation, and should be conducted at specialized centers with requisite expertise and backup [186,197]. A recent 2024 review by Nakai et al. indicates that after endoscopic or percutaneous draining of WON, a step-up method is used for necrosectomy, using either DEN or video-assisted retroperitoneal debridement [198]. The optimal timing for performing direct endoscopic necrosectomy in cases with necrotizing pancreatitis continues to be a matter of active discussion. In an international consensus survey, 86.4% of endoscopists recommended avoiding performing a DEN immediately (during the same session), while expert views on the optimal delay ranged from 3 to 21 days [199].

Successful studies have been published on the combination of percutaneous catheter drainage and endoscopic necrosectomy, which offers the benefits of both procedures (see Table 4), as follows: extensive accessibility; transperitoneal and/or retroperitoneal access to all abdominal locations; capability to insert, extract, and substitute multiple catheters for PCD and internal drainage; and prevention of external fistulae through the endoscopic technique [163,200,201]. In comparing the two procedures (endoscopic vs. PCD), it has been shown that patients in the PCD group had prolonged resolution times, a lower rate of normalization of SIRS parameters, increased need for surgical intervention, and a higher incidence of external pancreatic fistula [202,203].

##### Minimally Invasive Surgery

Minimally invasive surgery (MIS) has demonstrated reduced activation of the inflammatory response compared to open surgery, and experimental evidence indicates that local sepsis and the inflammatory response may be diminished with minimally invasive techniques [204,205]. In this context, the pancreatic necrosis debridement approach by a minimally invasive technique was investigated as a potentially better alternative to open surgery (see Table 6).

The Glasgow Group reported one of the initial experiences in this context by employing a combination of CT-guided drainage and an operative nephroscope, yielding promising outcomes [206]. Notwithstanding numerous subsequent publications [207,208,209] on this subject, the pivotal moment for the widespread acceptance of step-up approach with initial minimally invasive techniques for necrotizing pancreatitis was the PANTER study (Pancreatitis, Necrosectomy versus Step Up Approach) presented by the Dutch Pancreatitis Study Group [210]. This randomized controlled trial compared upfront open necrosectomy with continuous lavage (45 patients) to a step-up treatment (43 patients) that involved first percutaneous or endoscopic drainage, followed by video-assisted retroperitoneal debridement (VARD), if necessary. The efficacy of the step-up approach was evaluated using the primary endpoint, a composite of severe complications (new-onset multiple-organ failure or systemic complications, visceral organ perforation, enterocutaneous fistula, or hemorrhage) or mortality; this occurred in 69% of participants undergoing open necrosectomy and 40% of those receiving the step-up approach (*p* = 0.006).

The main indication for VARD remains central distribution of necrosis that extends down into the left paracolic gutter. The video-assisted retroperitoneal debridement technique starts with the image-guided placement of a percutaneous catheter into the retroperitoneal peripancreatic collection through the left flank. In instances where resolution is unattained, a surgical intervention is considered. The pathway created by the previously installed drain functions as the access route to the retroperitoneal compartment for intracavitary videoscopic necrosectomy. This treatment is conducted utilizing standard laparoscopic equipment under direct view with a laparoscope (0 or 30°) [211].

Complications with step-up therapy, including hemorrhage, intestinal fistula, and thrombosis, must be considered, along with concomitant concerns, such as the necessity of addressing the disconnected pancreatic duct syndrome [212,213].

The recently proposed use of real-time intraoperative near-infrared indocyanine green (ICG) fluorescence imaging during VARD (ICG-guided VARD) facilitates the visibility of surrounding well-perfused normal tissues, offering a distinct separation surface during debridement and mitigating the risk of vascular or intestinal injury [214].

#### 5.2.3. Open Surgery

Traditionally, open pancreatic necrosectomy was the primary surgical intervention for patients with infected pancreatic necrosis [215]. Historically, it was often performed during the early phase of the disease, before the necrosis became walled off, through a large-incision laparotomy, and it was aimed to remove as much infected necrotic tissue as possible to control the source of infection. However, this approach frequently resulted in the dissemination of infection and a secondary proinflammatory response, particularly in debilitated patients, leading to high mortality and morbidities like secondary organ failure, incisional hernia, and enteric and pancreatic fistula [216,217].

Despite initial inconsistent descriptions regarding delayed open pancreatic debridement [218,219], it was Buchler’s group who advocated for the postponement of pancreatic necrosectomy due to improved outcomes [220]. Early mortality in patients with severe acute pancreatitis is now less common, primarily due to advancements in intensive care treatment. Consequently, as noted by Beger, a subset of patients can be identified as suitable for the step-up approach, supporting the case for early surgical intervention in specific individuals [13].

In the last years, the surgical management of infected or complicated pancreatic necrosis has undergone a substantial shift from traditional open pancreatic necrosectomy to a minimally invasive step-up approach (see Table 7), which, compared with open pancreatic necrosectomy, has demonstrated superior short- and long-term clinical outcomes, as well as a lower incidence of complications [210,218,219]. Pancreatic necrosectomy with a minimally invasive step-up approach (see Table 5), compared with an open pancreatic necrosectomy, has demonstrated superior short- and long-term clinical outcomes, as well as a lower incidence of complications [210,221,222].

Although the minimally invasive step-up approach has proven effective, open pancreatic necrosectomy remains indispensable in specific critical situations. It can be a life-saving intervention for patients with worsening multiple organ dysfunction syndrome due to abdominal compartment syndrome [227]. Additionally, for patients who do not respond to minimally invasive step-up approach interventions or develop severe complications, open pancreatic necrosectomy serves as the definitive treatment [228]. Furthermore, in selected cases, primary open pancreatic necrosectomy showed favorable outcomes [226]. Therefore, open pancreatic necrosectomy remains a vital component of the surgical management strategy for severe retroperitoneal infections.

From a technical perspective, open surgical necrosectomy involves a median laparotomy, followed by entry into the omental bursa and removal of necrotic tissue. Blunt dissection is recommended because sharp dissection increases the risk of removing normal tissue, bleeding, and iatrogenic fistula formation with the bowel or pancreatic duct [229]. Multiple drains will be placed in the remaining cavity for subsequent lavage and for the control of a possible fistula. Four techniques for postoperative management of the pancreatic and retroperitoneal areas were described, as follows: open marsupialization [230,231], closed drainage [232], postoperative lavage [233,234], and staged reoperative approach [235,236].

Open surgical necrosectomy might offer the advantage of ensuring more complete removal of necrotic tissue while also allowing for the simultaneous management of multiple associated complications. However, due to its invasive nature, this procedure is typically reserved for patients in whom minimally invasive techniques have failed.

#### 5.2.4. Surgical Management of Complications

##### Abdominal Compartment Syndrome

Characterized by an elevation in intra-abdominal pressure exceeding 20 mm Hg, abdominal compartment syndrome is a highly concerning complication of acute pancreatitis that necessitates urgent surgical intervention for decompression [227]. Laparotomy for decompression should be contemplated for patients exhibiting persistent organ failure resulting from intra-abdominal hypertension (intra-abdominal pressure > 12 mmHg). The timing of intervention is paramount. Mentula et al. indicated that patients with severe acute pancreatitis experienced worse outcomes when decompression occurred more than four days post-ICU admission [237]. Surgical decompression results in an open abdomen, which poses risks including substantial fluid loss, infection, enterocutaneous fistulas, and ventral hernia. A complete midline laparotomy, spanning from the xiphoid process to the pubis, is the most frequently utilized technique; however, less invasive alternatives such as subcutaneous linea alba fasciotomy have also been published [238,239].

##### Hemorrhage

Pancreatic necrosis interventions, such as percutaneous drains, endoscopic necrosectomy and surgical necrosectomy, may lead to arterial pseudoaneurysms, predominantly affecting the splenic arteries (35–50%), as well as the gastroduodenal and pancreaticoduodenal arteries (20–25%) [194,240]. The mortality and morbidity associated with a rupture of this type of aneurysm ranges from 34% to 52% [241]. Management entails trans-arterial embolization, with or without stenting, to avert collateral backflow; otherwise, surgical intervention is mandatory [242].

##### Disconnected Pancreatic Duct Syndrome

Disconnected pancreatic duct syndrome is a significant but frequently overlooked complication of necrotizing pancreatitis, affecting 30–50% of cases [243,244,245]. It results from complete transection of the main pancreatic duct due to central necrosis, typically at the pancreatic neck, creating a disconnect between upstream viable pancreatic tissue and the gastrointestinal tract. Traditional treatment of disconnected pancreatic duct syndrome after necrosectomy includes surgical resection of the upstream gland. Alternatively, if the upstream pancreatic duct is sufficiently large, Roux-en-Y pancreatojejunostomy may be performed [246].

##### Colonic and Enteric Fistula

In severe acute pancreatitis, pancreatic enzymes and necrosis usually spread, causing enteric necrosis, especially colonic necrosis. In 17% to 19% of cases, colonic fistulas develop, which raises mortality and frequently requires colonic resection. Gao et al. showed improved outcomes using a step-up approach in the treatment of colonic fistulas in a study involving 711 patients, with percutaneous drainage resolving 47% of cases [247]. Additionally, patients who underwent percutaneous drainage had a lower mortality rate (19% versus 37%) than those who underwent surgery.

## 6. Conclusions

The management of pancreatic necrosis is a challenging and emerging area within acute pancreatitis care. Optimal results depend on early risk assessment, prompt referral to specialized facilities, and a multidisciplinary strategy customized to specific patient characteristics. Preventive treatments designed to diminish the severity of the disease (such as early fluid resuscitation, nutritional support, and the avoidance of unnecessary procedures) can alleviate progression to necrosis and related consequences. The timing of intervention is a crucial factor in therapeutic success. The interventional care of pancreatic necrosis has transitioned to a delayed and minimally invasive approach, informed by patient-specific factors and collaborative decision making among specialists. Additional comparative research and long-term outcome evaluations are necessary to enhance treatment protocols and improve patient care.

## Figures and Tables

**Table 1:** Selection of several severity prediction options in acute pancreatitis.

System/Biomarker	Cut-Off	Sensitivity	Specificity
Systemic Inflammatory Response System (SIRS) [41,42,43]	>2 points	55%	88%
APACHE score [44,45,46]	≥8 points	93.3%	85.7%
Blood Urea Nitrogen [47,48,49]	≥25 mg/dL	95.7%	97.4%
Bedside Index of Acute Pancreatitis (BISAP) [50,51,52]	≥2 points	73.5%	82.4%

**Table 3:** Summary of studies that evaluated the impact of prophylactic antibiotics in acute pancreatitis.

Author, Year	Intervention/Control	N	Age, Years	Female, N (%)	Antibiotic	Duration of Administration	Infected Pancreatic Necrosis	Non Pancreatic Infection	Positive Blood Culture	Fungal Infection	Need for Surgery	Mortality, N (%)
Poropat 2019 [118]	Intervention	49	74	41%	Imipenem	7–21 days	3. (6.12%)	10 (20.40%)	1 (2.04%)	1 (2.04%)	0	7 (14.28%)
	Control	49	74	45%	Placebo		2 (4.08%)	10 (20.40%)	1 (2.04%)	2 (4.08%)	0	8 (16.32%)
Garrcia-Barrasa 2009 [119]	Intervention	22	59.5	36%	Ciprofloxacin	10 days	8 (36.36%)	6 (27.27%)	3 (13.63%)	NA	11 (50%)	4 (18.18%)
	Control	19	67	21%	None		8 (42.10%)	8 (42.10%)	2 (10.52%)	NA	8 (42.10%)	2 (10.52%)
Xue 2009 [120]	Intervention	29	48.4 ± 15.1	52%	Imipenem-cilastatin	7–14 days	8 (27.58%)	18 (62.06%)	6 (20.68%)	NA	8 (27.58%)	3 (10.34%)
	Control	27	47.5 ± 12.3	48%	None		10 (37.03%)	15 (55.55%)	7 (25.92%)	NA	9 (33.33%)	4 (14.81%)
Dellinger 2007 [121]	Intervention	50	NA	36%	Meropenem	7–21 days	9 (18%)	16 (32%)	NA	2 (4%)	13 (26%)	10 (20%)
	Control	50	NA	24%	Placebo		6 (12%)	24 (48%)	NA	1 (2%)	10 (20%)	9 (18%)
Rokke 2007 [122]	Intervention	36	60	36%	Imipenem	5–7 days	3 (8.33%)	3 (8.33%)	NA	0	3 (8.33%)	3 (8.33%)
	Control	37	57	30%	None		7 (12.28%)	12 (32.43%)	NA	3 (8.10%)	3 (8.10%)	4 (8.10%)
Isenmann 2004 [123]	Intervention	58	47.9	26%	Ciprofloxacin + Metronidazole	21 days	7 (12.06%)	13 (22.41%)	NA	2 (3.44%)	10 (1.72%)	3 (5.17%)
	Control	56	45.6	21%	Placebo		5 (8.92%)	13 (23.21%)	NA	1 (1.78%)	6 (10.71%)	4 (7.14%)
Nordback 2001 [124]	Intervention	25	47.7	8%	Imipenem cilastatin	14 days	1 (4%)	4 (16%)	NA	1 (4%)	2 (8%)	2 (8%)
	Control	33	46.7	15%	None		6 (18.18%)	1 (3.03%)	NA	0	5 (15.15%)	5 (15.15%)

**Table 4:** Indications for interventions in acute necrotizing pancreatitis (excluding biliary component).

1. Infected pancreatic necrosis unresponsive to conservative management exhibiting increasing systemic sepsis, hemodynamic instability or organ failure.
2. Sterile pancreatic necrosis with the extent of necrosis of more than 50% of the pancreatic parenchyma, rapid deterioration, or persistent organ failure.
3. Failure of conservative management regarding symptoms (abdominal pain, vomiting, or other symptoms) or fluid collections.
4. Complications associated with acute necrotizing pancreatitis (abdominal compartment syndrome, pseudoaneurysm, bleeding, disconnected pancreatic duct syndrome, perforation, obstruction, or fistula).

**Table 5:** Summary of studies comparing early versus late intervention in acute necrotizing pancreatitis.

Name of Study	Group	Number of Patients	Endoscopic	Percutaneous	Surgical	Organ Failure	Length of Hospital Stay	Mortality
Guo 2014 [146]	Early	136	0	22	114	61	NA	21%
	Delayed	27	0	15	72	21	NA	10%
Woo 2017 [147]	Early	7	NA	NA	NA	NA	NA	0%
	Delayed	23	NA	NA	NA	NA	NA	17%
Mallick 2018 [148]	Early	258	0	258	0	98	NA	19%
	Delayed	117	0	117	0	19	NA	14%
Chanatarojanasiri 2018 [149]	Early	12	12	0	0	NA	27.5 days	8.33%
	Delayed	23	23	0	0	NA	31 days	7.70%
Trikudanathan 2018 [150]	Early	76	49	24	5	NA	37 days	13.20%
	Delayed	117	95	21	7	NA	26 days	4.30%
Oblizajek 2020 [151]	Early	19	19	0	0	NA	26 days	19.00%
	Delayed	19	19	0	0	NA	6 days	5.00%
Ganaie 2021 [152]	Early	24	0	24	0	NA	NA	NA
	Delayed	16	0	16	0	NA	NA	NA
Gupta 2021 [153]	Early	90	0	90	0	NA	NA	NA
	Delayed	54	0	54	0	NA	NA	NA
Khan 2021 [154]	Early	16	16	0	0	NA	NA	5.23%
	Delayed	172	172	0	0	NA	NA	18.75%
Rana 2021 [155]	Early	34	34	0	0	15	NA	5.70%
	Delayed	136	136	0	0	0	NA	0.00%
Boxhoorn 2021 [156]	Early	55	NA	NA	NA	13	59 days	13%
	Delayed	49	NA	NA	NA	8	51 days	10%
Jagielski 2022 [157]	Early	25	25	0	0	NA	NA	4%
	Delayed	46	46	0	0	NA	NA	4%
Lu 2022 [158]	Early	43	0	43	0	19	40.28 (median)	14%
	Delayed	55	0	55	0	10	47.76 (median)	11%
Zhang 2022 [159]	Early	100	0	100	NA	57	43 days	35%
	Delayed	31	0	31	NA	14	40 days	32.30%
Bhatia 2023 [160]	Early	74	0	74	0	0	28 days	24.30%
	Delayed	74	0	74	0	0	29.4 days	18.90%
Ali 2024 [161]	Early	349	NA	NA	NA	NA	6.0 (median)	1.10%
	Delayed	375	NA	NA	NA	NA	16.0 (median)	4.30%

**Table 6:** Comparative overview of indications, advantages, and disadvantages of therapeutic modalities for acute necrotizing pancreatitis.

	Indications	Advantages	Disadvantages
Endoscopic management	Infected WON Symptomatic sterile WON Progressive clinical deterioration	Minimal invasiveness Lower mortality Avoiding digestive stomas Possibility of repeated procedures Effective infection control Shorter hospital stay	Local complications (stent migration, bleeding, perforations) Limited availability and expertise in less equipped centers Low efficiency in non-organized necrosis, requiring complementary procedures The need for multiple procedures Risk of recurrence of collections High costs
Percutaneous management	First step in a step-up approach Infected pancreatic necrosis Retroperitoneal or poorly accessible collection for endoscopic drainage Hemodynamically unstable patients-as a first step Failed or contraindicated endoscopic drainage	Minimal invasiveness Lower mortality within the step up approach Possibility of use in early stages Technique available in most centers Allows drainage of multiple collections Can be used as a bridge to other therapies	Limited efficacy in solid necrosis Need for long-term drainage Risk of pancreatico-cutaneous fistula Possibility of incomplete drainage Procedure-related complications (bleeding, secondary peritonitis or catheter migration) Frequent recurrence of collections
Surgical management	Infected necrotizing pancreatitis, when minimally invasive approaches fail Progressive clinical deterioration Extensive necrosis with complications (ischemia, perforation, bleeding) Obstructive complications Failure of percutaneous or endoscopic drainage	Removal of necrotic tissue Infection control Improvement of drainage Resolving associated complications Possibility of association with other surgical procedures Effectiveness in cases refractory to minimally invasive treatment	High mortality and morbidity Risk of pancreatic insufficiency Increased length of hospital stay Postoperative complications (incisional hernias, pancreatic fistulas, new onset diabetes)

**Table 7:** Summary of studies comparing open pancreatic necrosectomy versus minimally invasive techniques in acute necrotizing pancreatitis.

First Author, Year, Type of Study	Number of Patients	Median Age	Male n (%)	APACHE	Time of Intervention (Median)	Organ Failure	Bleeding	Perforation of a Visceral Organ	Pancreatic Fistula	Death n (%)	Long Term Complications
van Santvoort 2010 (PANTER), prospective [210]											
OPN	45	57.4	33 (72%)	15	NA	18	10	10	17	7 (16%)	43
Minimally invasive step-up approach	43	57.6	31 (73%)	14		5	7	6	12	8 (19%)	13
Gomatos 2016, retrospective [223]											
OPN	120	58.5	86 (71.7%)	8	24 days	56	NA	NA	NA	28 (23.3%)	NA
MARPN	274	59	172 (62.8%)	8	29.5 days	42				42 (15.3%)	
van Brunschot 2018, retrospective [222]											
OPN	47	60	29 (61.7%)	10	41 days	7	10	8	13	6 (12.7%)	14
Endoscopy	51	63	34 (66.6%)	9	39.5 days	9	11	4	2	9 (17.6%)	16
Martinez 2019, retrospective [224]											
OPN	34	55.9	24 (70.6%)		10.5 days	16	NA	NA	NA	7 (20.6%)	10
Endoscopy	22	53.3	16 (72.7%)		38 days	2				0	15
Luckhurst 2020, retrospective [225]											
OPN	88	56	64 (73%)	9	42 days	NA	8	NA	61	8 (9%)	35
Minimally invasive step-up approach	91	54	62 (68%)	10	36 days		17		59	2 (2%)	42
Ning 2024, prospective [226]											
OPN	75	48	56 (74.6%)		NA	32	26	25	33	27 (36%)	NA
Minimally invasive step-up approach	245	48	178 (72.7%)			76	42	21	107	47 (19.2%)	

OPN—open pancreatic necrosectomy; MARPN—minimal access retroperitoneal pancreatic necrosectomy.
